# 2-Amino-6-methyl-1,3-benzothia­zole–deca­nedioic acid (2/1)

**DOI:** 10.1107/S1600536809031857

**Published:** 2009-08-19

**Authors:** Xiang-Jun Shi, Zhi-Chao Wang, Qiang Chen, Xiao-Jun Zhao

**Affiliations:** a College of Chemistry and Life Science, Tianjin Key Laboratory of Structure and Performance of Functional Molecule, Tianjin Normal University, Tianjin 300387, People’s Republic of China

## Abstract

Co-crystallization of 2-amino-6-methyl-1,3-­benzothia­zole with deca­nedioic acid under hydro­thermal conditions afforded the title 2:1 co-crystal, 2C_8_H_8_N_2_S·C_10_H_18_O_4_. The deca­nedioic acid mol­ecule is located on an inversion centre. In the crystal, inter­molecular N—H⋯O and O—H⋯O hydrogen bonds connect the components into a two-dimensional wave-like layer structure extending parallel to (100).

## Related literature

For mol­ecular self-assembly and crystal engineering, see: Sun & Cui (2008 ▶); Hunter (1993 ▶); Yang *et al.* (2005 ▶). For the solid structures and properties of metal complexes of amino­benzothia­zole and its derivatives, see: Lynch *et al.* (1998 ▶, 1999 ▶); Sun & Cui (2008 ▶); Popović *et al.* (2002 ▶); Antiñolo *et al.* (2007 ▶); Dong *et al.* (2002 ▶); Chen *et al.* (2008 ▶); Zhang *et al.* (2009 ▶). For the structures of deca­nedioic acid-based metal complexes and co-crystals, see: Xian *et al.* (2009 ▶); Braga *et al.* (2006 ▶); Aakeröy *et al.* (2007 ▶).
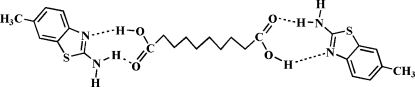


## Experimental

### 

#### Crystal data

2C_8_H_8_N_2_S·C_10_H_18_O_4_
*M* *_r_* = 530.71
Monoclinic, 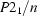

*a* = 5.3791 (5) Å
*b* = 21.822 (2) Å
*c* = 11.9431 (11) Å
β = 91.6660 (10)°
*V* = 1401.3 (2) Å^3^
*Z* = 2
Mo *K*α radiation
μ = 0.23 mm^−1^
*T* = 293 K
0.32 × 0.24 × 0.22 mm

#### Data collection

Bruker APEXII CCD area-detector diffractometer
Absorption correction: multi-scan (*SADABS*; Sheldrick, 1996 ▶) *T*_min_ = 0.931, *T*_max_ = 0.952
7545 measured reflections
2470 independent reflections
1822 reflections with *I* > 2σ(*I*)
*R*_int_ = 0.024

#### Refinement

*R*[*F* ^2^ > 2σ(*F* ^2^)] = 0.039
*wR*(*F* ^2^) = 0.109
*S* = 1.05
2470 reflections
164 parameters
H-atom parameters constrained
Δρ_max_ = 0.23 e Å^−3^
Δρ_min_ = −0.26 e Å^−3^

### 

Data collection: *APEX2* (Bruker, 2003 ▶); cell refinement: *SAINT* (Bruker, 2001 ▶); data reduction: *SAINT*; program(s) used to solve structure: *SHELXS97* (Sheldrick, 2008 ▶); program(s) used to refine structure: *SHELXL97* (Sheldrick, 2008 ▶); molecular graphics: *SHELXTL* (Sheldrick, 2008 ▶) and *DIAMOND* (Brandenburg & Berndt, 1999 ▶); software used to prepare material for publication: *SHELXL97*.

## Supplementary Material

Crystal structure: contains datablocks I, global. DOI: 10.1107/S1600536809031857/bt5031sup1.cif

Structure factors: contains datablocks I. DOI: 10.1107/S1600536809031857/bt5031Isup2.hkl

Additional supplementary materials:  crystallographic information; 3D view; checkCIF report

## Figures and Tables

**Table 1:** Hydrogen-bond geometry (Å, °)

*D*—H⋯*A*	*D*—H	H⋯*A*	*D*⋯*A*	*D*—H⋯*A*
O1—H1⋯N1	0.82	1.78	2.597 (2)	171
N2—H2*A*⋯O2	0.86	2.09	2.914 (2)	161
N2—H2*B*⋯O1^i^	0.86	2.14	2.954 (2)	157

Symmetry code: (i) 
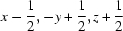
.

**Fig. 1.**
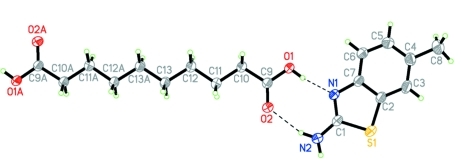
The molecular structure of (I). Displacement ellipsoids are drawnat the 30% probability level. The dashed lines indicate intermolecular hydrogen bonds.[Symmetry code: (A) 4 – x, 1 – y, – z]

**Fig. 2.**
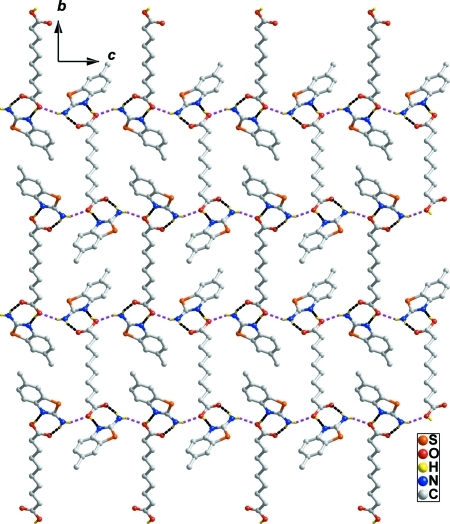
The two-dimensional layer of (I) formed by N–H···O and O–H ···O hydrogen bonding interactions.

## supplementary crystallographic information

### Comment

During the past decades, molecular self-/assembly by classical coordination bonds and/or intermolecular non-covalent interactions such as hydrogen-bonding, π···π stacking, electrostatic interactions and so on, has been becoming more and more attractive in biology, biochemistry and new material fields (Sun *et al.*, 2008; Hunter, 1993; Yang *et al.*, 2005).

Acting as one of the excellent building blocks with multiple hydrogen-bonding sites and metal ion binding donors, aminobenzothiazole and its derivatives have been extensively utilized in the new materials, biochemistry and agriculture chemistry, due to the lower toxicity, high biological activity as well as excellent chemical reactivity (Lynch *et al.*, 1998; Lynch *et al.*, 1999; Sun *et al.*, 2008; Popović *et al.*, 2002;   Antiñolo *et al.*, 2007; Dong *et al.*, 2002; Chen *et al.*, 2008). On the other hand, the long decanedioic acid with adjustable deprotonated form and flexible aliphatic chain has also exhibited novel functions such as dianion templating (Xian *et al.*, 2009) and heterosynthons with nitrogen-containing compounds (Braga *et al.*, 2006; Aakeröy *et al.*, 2007) in the fields of metal complexes and molecular co-crystals.

Thus, as a continuation of molecular assembly behavior in the solid state, in the present paper, the rigid 2-amino-6-methyl-1,3-benzothiazole (Ambt) and flexible decanedioic acid were selected as building blocks to cocrystallize. As a result, an intermolecular hydrogen bonded adduct, (I), was obtained under the hydrothermal conditions.

As shown in Fig. 1, the asymmetric unit of (I) comprises one neutral Ambt molecule with no crystallographically imposed symmetry and half a decanedioic acid located on  a centre of inversion. Obviously, no proton transfer was observed for the neutral cocrystal, which is much different from the 2-aminobenzothiazolium 2,4-dicarboxybenzoate monohydrate (Zhang *et al.*, 2009). The exocyclic amino group of Ambt is roughly coplanar with the benzothiazole ring. Similarily, the carboxylic residues of decanedioic acid are also co-planar with their long aliphatic chain. In the packing structure of I, two pairs of the intermolecuar O1—H1 ···N1 and N2—H2A ···O2 hydrogen-bonding interactions (Table 1) connect the two Ambt molecules and one decanedioic acid.

### Experimental

To an aqueous solution of Ambt (40.4 mg, 0.2 mmol) was slowly added an aqueous solution of decanedioic acid (20.2 mg, 0.1 mmol) with constant stirring. After further stirring for about ten minutes, the resulting mixture was sealed in a stainless steel vessel and heated at 140 *^o^*C for 3 days. After the mixture was cooled to room temperature at a rate of 5 *^o^*C / h, pale-yellow block-shaped crystals suitable for X-ray diffraction were obtained directly, washed with ethanol and dried in air.Yield: 34% based on Ambt. Anal. calcd for C_13_H_17_N_2_O_2_S: C, 58.84; H, 6.46; N, 10.56%. Found: C, 58.78; H, 6.56; N,10.70%.

### Refinement

H-atoms were located in difference maps, but were subsequently placed in calculated positions and treated as riding, with C—H = 0.93 Å, O—H = 0.82 Å, and N—H = 0.86 Å. all H atoms were allocated displacement parameters related to those of their parent atoms [*U*_iso_(H)] = 1.2 *U*_eq_ (C, N, O)

### Crystal data

**Table:**

2C_8_H_8_N_2_S·C_10_H_18_O_4_	*F*(000) = 564
*M**_r_* = 530.71	*D*_x_ = 1.258 Mg m^−^^3^
Monoclinic, *P*2_1_/*n*	Mo *K*α radiation, λ = 0.71073 Å
*a* = 5.3791 (5) Å	Cell parameters from 1765 reflections
*b* = 21.822 (2) Å	θ = 2.5–22.8°
*c* = 11.9431 (11) Å	µ = 0.23 mm^−^^1^
β = 91.666 (1)°	*T* = 293 K
*V* = 1401.3 (2) Å^3^	Block, pale-yellow
*Z* = 2	0.32 × 0.24 × 0.22 mm

### Data collection

**Table:**

Bruker APEXII CCD area-detector diffractometer	2470 independent reflections
Radiation source: fine-focus sealed tube	1822 reflections with *I* > 2σ(*I*)
graphite	*R*_int_ = 0.024
φ and ω scans	θ_max_ = 25.0°, θ_min_ = 1.9°
Absorption correction: multi-scan (*SADABS*; Sheldrick, 1996)	*h* = −6→6
*T*_min_ = 0.931, *T*_max_ = 0.952	*k* = −25→21
7545 measured reflections	*l* = −14→14

### Refinement

**Table:**

Refinement on *F*^2^	Primary atom site location: structure-invariant direct methods
Least-squares matrix: full	Secondary atom site location: difference Fourier map
*R*[*F*^2^ > 2σ(*F*^2^)] = 0.039	Hydrogen site location: inferred from neighbouring sites
*wR*(*F*^2^) = 0.109	H-atom parameters constrained
*S* = 1.05	*w* = 1/[σ^2^(*F*_o_^2^) + (0.0483*P*)^2^ + 0.3107*P*] where *P* = (*F*_o_^2^ + 2*F*_c_^2^)/3
2470 reflections	(Δ/σ)_max_ < 0.001
164 parameters	Δρ_max_ = 0.23 e Å^−^^3^
0 restraints	Δρ_min_ = −0.26 e Å^−^^3^

### Special details

**Table:**

Geometry. All e.s.d.'s (except the e.s.d. in the dihedral angle between two l.s. planes) are estimated using the full covariance matrix. The cell e.s.d.'s are taken into account individually in the estimation of e.s.d.'s in distances, angles and torsion angles; correlations between e.s.d.'s in cell parameters are only used when they are defined by crystal symmetry. An approximate (isotropic) treatment of cell e.s.d.'s is used for estimating e.s.d.'s involving l.s. planes.
Refinement. Refinement of *F*^2^ against ALL reflections. The weighted *R*-factor *wR* and goodness of fit *S* are based on *F*^2^, conventional *R*-factors *R* are based on *F*, with *F* set to zero for negative *F*^2^. The threshold expression of *F*^2^ > σ(*F*^2^) is used only for calculating *R*-factors(gt) *etc*. and is not relevant to the choice of reflections for refinement. *R*-factors based on *F*^2^ are statistically about twice as large as those based on *F*, and *R*- factors based on ALL data will be even larger.

### Fractional atomic coordinates and isotropic or equivalent isotropic displacement parameters (Å^2^)

**Table:**

	*x*	*y*	*z*	*U*_iso_*/*U*_eq_	
S1	0.47667 (11)	0.17050 (3)	0.20230 (5)	0.0647 (2)	
O1	1.1513 (3)	0.27012 (7)	−0.03449 (11)	0.0595 (4)	
H1	1.0456	0.2531	0.0026	0.089*	
O2	1.2105 (3)	0.32343 (7)	0.12223 (12)	0.0662 (4)	
N1	0.8009 (3)	0.21067 (7)	0.06387 (13)	0.0502 (4)	
N2	0.8217 (3)	0.25824 (8)	0.23838 (14)	0.0628 (5)	
H2A	0.9420	0.2815	0.2189	0.075*	
H2B	0.7643	0.2610	0.3046	0.075*	
C1	0.7248 (4)	0.21790 (9)	0.16608 (16)	0.0495 (5)	
C2	0.4781 (4)	0.13827 (9)	0.06865 (17)	0.0536 (5)	
C3	0.3269 (4)	0.09285 (10)	0.02272 (19)	0.0658 (6)	
H3A	0.2024	0.0754	0.0648	0.079*	
C4	0.3619 (4)	0.07353 (10)	−0.0861 (2)	0.0647 (6)	
C5	0.5474 (4)	0.10135 (11)	−0.14663 (18)	0.0650 (6)	
H5A	0.5703	0.0889	−0.2201	0.078*	
C6	0.6984 (4)	0.14661 (10)	−0.10228 (17)	0.0599 (6)	
H6A	0.8214	0.1643	−0.1449	0.072*	
C7	0.6645 (4)	0.16541 (9)	0.00673 (16)	0.0488 (5)	
C8	0.1999 (6)	0.02374 (13)	−0.1383 (2)	0.0920 (9)	
H8A	0.0819	0.0101	−0.0850	0.138*	
H8B	0.3023	−0.0101	−0.1597	0.138*	
H8C	0.1130	0.0397	−0.2033	0.138*	
C9	1.2624 (4)	0.31276 (9)	0.02632 (16)	0.0469 (5)	
C10	1.4559 (4)	0.34752 (9)	−0.03539 (16)	0.0511 (5)	
H10A	1.3758	0.3681	−0.0987	0.061*	
H10B	1.5740	0.3185	−0.0648	0.061*	
C11	1.5968 (4)	0.39443 (9)	0.03408 (16)	0.0509 (5)	
H11A	1.6931	0.3735	0.0924	0.061*	
H11B	1.4788	0.4210	0.0701	0.061*	
C12	1.7698 (4)	0.43316 (9)	−0.03430 (17)	0.0517 (5)	
H12A	1.8855	0.4064	−0.0713	0.062*	
H12B	1.6726	0.4544	−0.0920	0.062*	
C13	1.9159 (4)	0.47985 (9)	0.03387 (16)	0.0511 (5)	
H13A	2.0164	0.4585	0.0901	0.061*	
H13B	1.8000	0.5058	0.0727	0.061*	

### Atomic displacement parameters (Å^2^)

**Table:**

	*U*^11^	*U*^22^	*U*^33^	*U*^12^	*U*^13^	*U*^23^
S1	0.0678 (4)	0.0744 (4)	0.0529 (3)	−0.0197 (3)	0.0191 (3)	0.0042 (3)
O1	0.0673 (10)	0.0637 (9)	0.0484 (8)	−0.0271 (7)	0.0198 (7)	−0.0071 (7)
O2	0.0763 (11)	0.0750 (10)	0.0484 (8)	−0.0275 (8)	0.0207 (7)	−0.0107 (7)
N1	0.0517 (10)	0.0565 (10)	0.0428 (9)	−0.0120 (8)	0.0086 (7)	0.0036 (7)
N2	0.0740 (13)	0.0691 (12)	0.0460 (9)	−0.0193 (10)	0.0129 (9)	−0.0042 (9)
C1	0.0512 (12)	0.0513 (11)	0.0464 (11)	−0.0036 (9)	0.0074 (9)	0.0084 (9)
C2	0.0538 (12)	0.0549 (12)	0.0524 (11)	−0.0087 (10)	0.0061 (9)	0.0091 (10)
C3	0.0606 (14)	0.0675 (14)	0.0695 (14)	−0.0214 (12)	0.0070 (11)	0.0113 (12)
C4	0.0671 (15)	0.0618 (13)	0.0646 (14)	−0.0132 (12)	−0.0080 (11)	0.0024 (11)
C5	0.0712 (15)	0.0740 (15)	0.0497 (12)	−0.0096 (12)	−0.0002 (11)	−0.0010 (11)
C6	0.0615 (14)	0.0691 (14)	0.0495 (12)	−0.0136 (11)	0.0063 (10)	0.0034 (10)
C7	0.0482 (12)	0.0515 (11)	0.0465 (11)	−0.0050 (9)	0.0012 (9)	0.0087 (9)
C8	0.097 (2)	0.0889 (19)	0.0893 (18)	−0.0331 (17)	−0.0044 (16)	−0.0102 (16)
C9	0.0476 (12)	0.0457 (11)	0.0477 (11)	−0.0043 (9)	0.0069 (9)	0.0008 (9)
C10	0.0518 (12)	0.0497 (11)	0.0525 (12)	−0.0099 (9)	0.0122 (9)	−0.0013 (9)
C11	0.0491 (12)	0.0511 (12)	0.0527 (11)	−0.0076 (9)	0.0057 (9)	0.0029 (9)
C12	0.0474 (12)	0.0519 (12)	0.0561 (12)	−0.0085 (9)	0.0078 (9)	−0.0001 (9)
C13	0.0482 (12)	0.0515 (12)	0.0538 (11)	−0.0069 (9)	0.0053 (9)	0.0040 (9)

### Geometric parameters (Å, °)

**Table:**

S1—C2	1.744 (2)	C6—C7	1.382 (3)
S1—C1	1.753 (2)	C6—H6A	0.9300
O1—C9	1.313 (2)	C8—H8A	0.9600
O1—H1	0.8200	C8—H8B	0.9600
O2—C9	1.209 (2)	C8—H8C	0.9600
N1—C1	1.308 (2)	C9—C10	1.499 (3)
N1—C7	1.397 (2)	C10—C11	1.508 (3)
N2—C1	1.329 (2)	C10—H10A	0.9700
N2—H2A	0.8600	C10—H10B	0.9700
N2—H2B	0.8600	C11—C12	1.514 (3)
C2—C3	1.385 (3)	C11—H11A	0.9700
C2—C7	1.395 (3)	C11—H11B	0.9700
C3—C4	1.385 (3)	C12—C13	1.510 (3)
C3—H3A	0.9300	C12—H12A	0.9700
C4—C5	1.388 (3)	C12—H12B	0.9700
C4—C8	1.516 (3)	C13—C13^i^	1.513 (4)
C5—C6	1.375 (3)	C13—H13A	0.9700
C5—H5A	0.9300	C13—H13B	0.9700
			
C2—S1—C1	89.34 (9)	C4—C8—H8C	109.5
C9—O1—H1	109.5	H8A—C8—H8C	109.5
C1—N1—C7	111.53 (16)	H8B—C8—H8C	109.5
C1—N2—H2A	120.0	O2—C9—O1	123.13 (17)
C1—N2—H2B	120.0	O2—C9—C10	123.42 (18)
H2A—N2—H2B	120.0	O1—C9—C10	113.44 (16)
N1—C1—N2	123.95 (18)	C9—C10—C11	114.71 (16)
N1—C1—S1	114.85 (15)	C9—C10—H10A	108.6
N2—C1—S1	121.18 (15)	C11—C10—H10A	108.6
C3—C2—C7	121.1 (2)	C9—C10—H10B	108.6
C3—C2—S1	129.25 (16)	C11—C10—H10B	108.6
C7—C2—S1	109.65 (15)	H10A—C10—H10B	107.6
C4—C3—C2	119.7 (2)	C10—C11—C12	112.90 (16)
C4—C3—H3A	120.2	C10—C11—H11A	109.0
C2—C3—H3A	120.2	C12—C11—H11A	109.0
C3—C4—C5	118.4 (2)	C10—C11—H11B	109.0
C3—C4—C8	120.8 (2)	C12—C11—H11B	109.0
C5—C4—C8	120.8 (2)	H11A—C11—H11B	107.8
C6—C5—C4	122.6 (2)	C13—C12—C11	113.84 (16)
C6—C5—H5A	118.7	C13—C12—H12A	108.8
C4—C5—H5A	118.7	C11—C12—H12A	108.8
C5—C6—C7	118.9 (2)	C13—C12—H12B	108.8
C5—C6—H6A	120.6	C11—C12—H12B	108.8
C7—C6—H6A	120.6	H12A—C12—H12B	107.7
C6—C7—C2	119.34 (19)	C12—C13—C13^i^	114.4 (2)
C6—C7—N1	126.03 (18)	C12—C13—H13A	108.7
C2—C7—N1	114.63 (17)	C13^i^—C13—H13A	108.7
C4—C8—H8A	109.5	C12—C13—H13B	108.7
C4—C8—H8B	109.5	C13^i^—C13—H13B	108.7
H8A—C8—H8B	109.5	H13A—C13—H13B	107.6
			
C7—N1—C1—N2	−179.62 (19)	C5—C6—C7—C2	−0.2 (3)
C7—N1—C1—S1	−0.5 (2)	C5—C6—C7—N1	−179.8 (2)
C2—S1—C1—N1	0.29 (17)	C3—C2—C7—C6	0.0 (3)
C2—S1—C1—N2	179.42 (18)	S1—C2—C7—C6	−179.96 (17)
C1—S1—C2—C3	−180.0 (2)	C3—C2—C7—N1	179.69 (19)
C1—S1—C2—C7	0.02 (16)	S1—C2—C7—N1	−0.3 (2)
C7—C2—C3—C4	0.5 (3)	C1—N1—C7—C6	−179.8 (2)
S1—C2—C3—C4	−179.45 (19)	C1—N1—C7—C2	0.5 (3)
C2—C3—C4—C5	−0.9 (4)	O2—C9—C10—C11	4.3 (3)
C2—C3—C4—C8	179.8 (2)	O1—C9—C10—C11	−176.76 (17)
C3—C4—C5—C6	0.8 (4)	C9—C10—C11—C12	−173.70 (17)
C8—C4—C5—C6	−179.9 (2)	C10—C11—C12—C13	−179.12 (17)
C4—C5—C6—C7	−0.2 (4)	C11—C12—C13—C13^i^	−178.3 (2)

Symmetry codes: (i) −*x*+4, −*y*+1, −*z*.

### Hydrogen-bond geometry (Å, °)

**Table:**

*D*—H···*A*	*D*—H	H···*A*	*D*···*A*	*D*—H···*A*
O1—H1···N1	0.82	1.78	2.597 (2)	171
N2—H2A···O2	0.86	2.09	2.914 (2)	161
N2—H2B···O1^ii^	0.86	2.14	2.954 (2)	157

Symmetry codes: (ii) *x*−1/2, −*y*+1/2, *z*+1/2.
